# Physicians’ assessment of the Bavarian drug-expenditure control system: a qualitative study

**DOI:** 10.1186/s12913-023-09844-3

**Published:** 2023-09-07

**Authors:** Nikoletta Zeschick, Julia Gollnick, Julia Muth, Franziska Hörbrand, Peter Killian, Norbert Donner-Banzhoff, Thomas Kühlein, Maria Sebastião

**Affiliations:** 1 Friedrich-Alexander-Universität Erlangen-Nürnberg (FAU), Allgemeinmedizinisches Institut, Universitätsstr. 29, 91054 Erlangen, Germany; 2 Abteilung Für Allgemeinmedizin, Philipps Universität Marburg, Präventive und Rehabilitative Medizin, Karl-Von-Frisch-Straße 4, 35032 Marburg, Germany; 3 Kassenärztliche Vereinigung Bayerns, Elsenheimerstraße 39, 80687 Munich, Germany

**Keywords:** Wirkstoffvereinbarung, Active substance agreement, Drug prescriptions, Economic efficiency, Drug costs

## Abstract

**Background:**

In 2014 a new system for drug expenditures, the Wirkstoffvereinbarung (WSV, *English*: Active substance agreement) was implemented in Bavaria. In pre-defined indication groups, economic prescription of medications shall be enabled based on the selection, quantity, and proportion of an individual drug. Ambulatory care physicians receive quarterly trend reports on their prescribing behavior. This study examines physicians’ perceptions of the WSV.

**Methods:**

Qualitative interviews (*n* = 20) and seven focus groups (*n* = 36) were conducted with ambulatory care physicians (e.g. general practitioners, cardiologists, pulmonologists). The methodology followed Qualitative Content Analysis.

**Results:**

Physicians generally accepted the necessity of prescribing economically. The majority of them rated the WSV positively and better than the previous system. As an improvement, they especially named timely feedback in form of easily understandable trend reports, encouraging self-reflection as well as allowing early control options. Problems perceived were drug discount contracts that were strongly criticized as leading to patients mixing up medications. Some perceived constraints of therapeutic freedom.

**Conclusions:**

The implementation of the WSV is mostly viewed positively by physicians. The restrictions of therapeutic freedom partially perceived might be met by improved information on the reasons why some drugs are rated as less economical than others.

**Trial registration number:**

Main ID: DRKS00019820 (German Register of Clinical Studies and World Health Organization).

**Supplementary Information:**

The online version contains supplementary material available at 10.1186/s12913-023-09844-3.

## Background

Drug expenditures are growing yearly and with € 615 per capita, Germany has in 2018 with 60% above the EU average the highest drug costs among EU member states [1]. In most EU member states, the costs of drugs are predominantly covered by government or compulsory insurance schemes, with public coverage being most generous in Germany [1]. The reasons for the increase in drug expenditures in the different European countries are similar and long-known [2]. This includes an increase in the proportion of older residents, the incidence and duration of chronic diseases, the continuing development of new drugs mostly with small additional benefits but far higher prices, and the rising expectations of patients and society regarding health [2]. At the same time, Germany is only in the average range in terms of health outcomes [3–5].

According to the efficiency principle, all health care must be sufficient, appropriate, and economical (§12 SGB V). To ensure the economic efficiency of healthcare, the sick funds, and the regional Associations of Statutory Health Insurance Physicians (ASHIPs) agree on regulations controlling the prescription of drugs (§84 und §106b SGB V). In Bavaria, this scheme is called “Wirkstoffvereinbarung” (WSV; *English*: Active Substance Agreement) [6].

Many German federal states have or had the so-called “Richtgrößenprüfung” (RGP; *English*: Prescribing Target Scheme) [7]. The RGP also existed in Bavaria, before WSV has been put in place. In the RGP, a certain annual volume of costs in € for drug prescriptions was set for each physician. If this was exceeded by 25% or more, the respective physician was at risk of sanctions. A practice was exempt, if particular practice characteristics could be demonstrated, such as a high number of multimorbid patients [7]. The threat of sanctions might contribute to the increasing unattractiveness of self-employment in the outpatient sector, with the consequence of a shortage of young physicians, especially in primary care [8]. The RGP had and has many weaknesses, foremost low transparency of regulations [9]. [9]. Moreover, the RGP is based on budgeting as cost containment. Studies could not show a clear effect of budgeting as cost containment [10]. Because of the shortcomings of the RGP, the Bavarian ASHIP, and the Bavarian sick funds implemented a new system in Bavaria by introducing the WSV in 2014. The WSV is a system that emphasizes the importance of aligning prescribed behaviors with economic efficiency principles. For example, when treating a particular medical condition, there may be multiple medications available that can achieve similar outcomes. The economic efficiency principle suggests that the medication that provides the best health outcome for the patient, while minimizing costs, should be chosen. This system is intended to prevent sanctions. The WSV enables economic prescriptions based on the selection of recommended drugs, their quantity, and the proportion of them prescribed in specific indication groups using recommended drug targets of generics, lead substances, and discount contracts for specific drugs [11]. Generics are considered an economic alternative to brand drugs with the same qualitative and quantitative composition of the drug compound, which become available after patent expiry. [12]. Lead substances are determined to be more economical for drugs with comparable efficacy [13]. Lead substances are active ingredients that are commonly used as reference substances or standards in the treatment of specific diseases or conditions in medicine. In the context of the WSV, lead substances are defined as central active ingredients that should be considered when selecting medications. The selection of lead substances is based on evidence-based recommendations and economic reasons. An example of a lead substance is the vitamin K antagonist Phenprocoumon.

Discount contracts are concluded between pharmaceutical companies and sick funds and have an additional financial savings potential [14]. If a patient has a prescription for a brand-name drug from a manufacturer other than the discount contractor, pharmacists are instructed to dispense the contractor's drug [15].

**Fig. 1:**
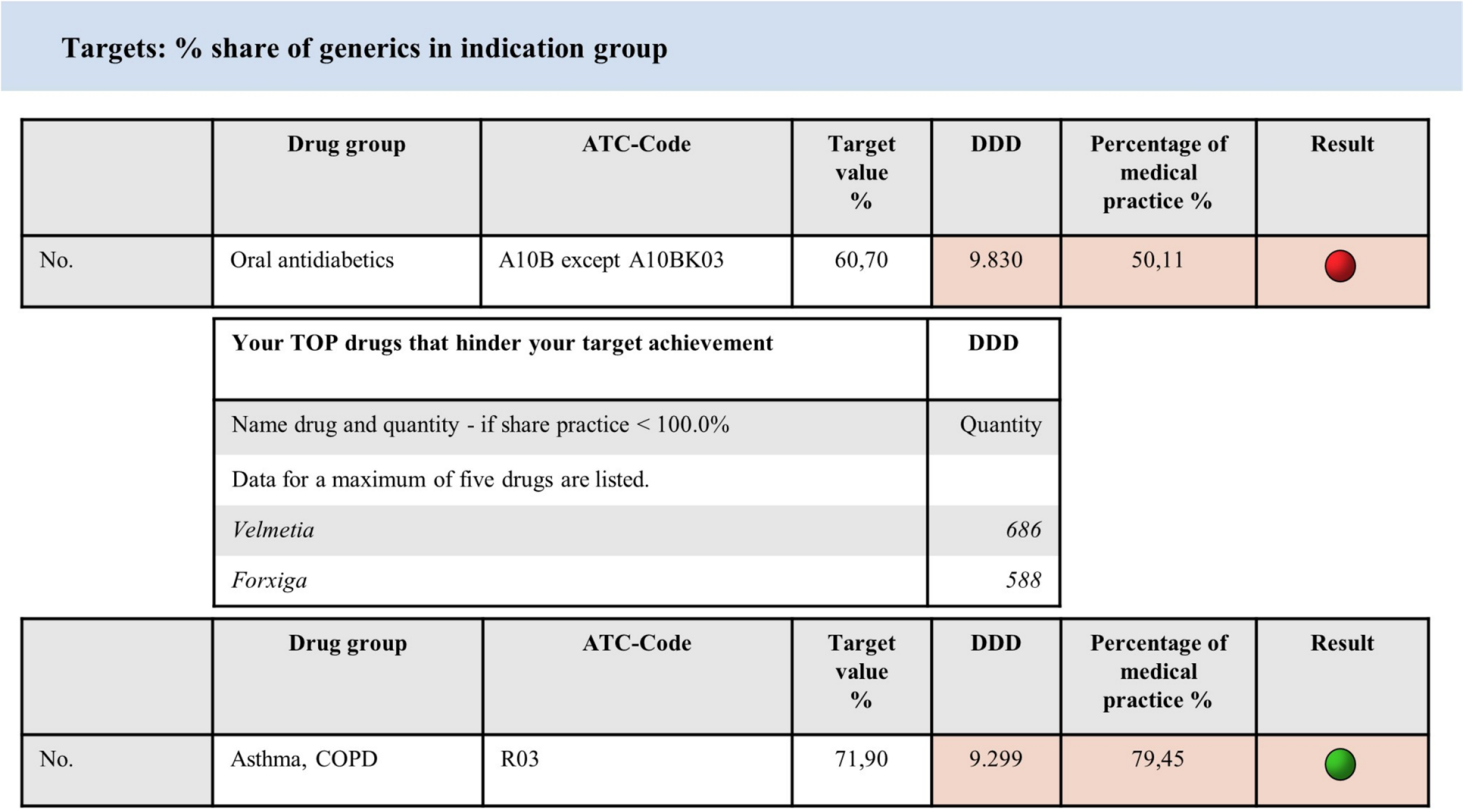
Excerpt from the WSV: Trend reports of the ASHIP, Bavaria displaying target achievements of individual indication groups [16]

For drugs covered by the WSV physicians receive a detailed quarterly trend report as early information on their prescribing behavior and their achievement of agreed target values. The information in the drug trend report is visualized by a traffic light system (green/yellow/red) that will help the physicians to see where the prescribing behavior can be adjusted to follow the idea of an economic drug prescription (Fig. 1).

**Fig. 2:**
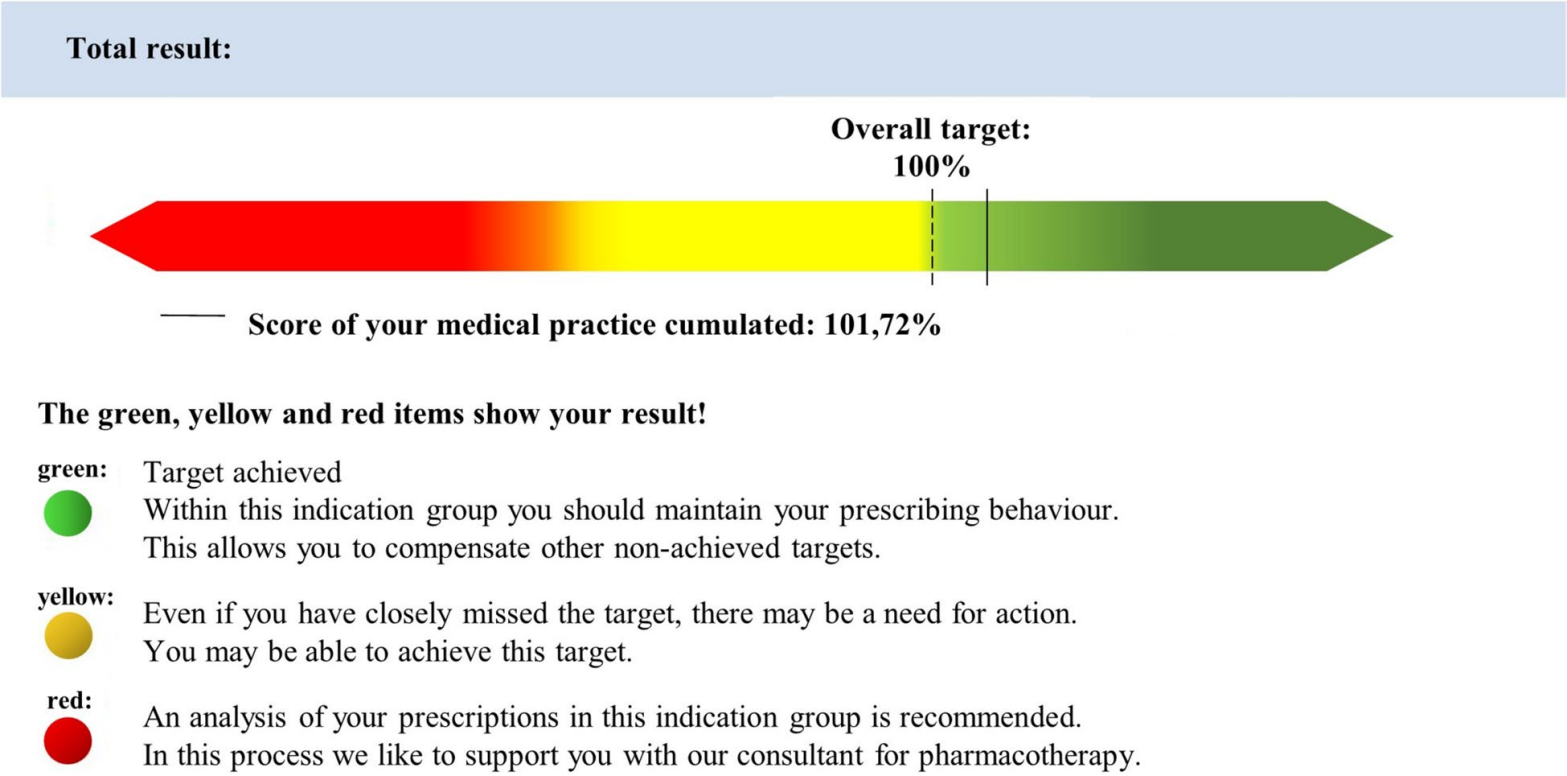
Excerpt from the WSV: Trend reports of the ASHIP, Bavaria displaying the total target achievement (cumulated) [16]

In addition, an overall result—the total target achievement—weighted by prescription volume is calculated for all prescribed indication groups. If the targets are met or exceeded, the target achievement is ≥ 100%, which exempts a medical practice from an audit for the respective quarter (Fig. 2). The WSV pursues the goal that physicians no longer have to worry about the costs of individual drugs, but they have to meet the targets.

The WSV was revised in 2017 (WSV2.0) and 2020 (WSV 3.0). The targets of the individual indication groups, for example, were adjusted in line with changes in the market e.g. discount agreements or patent expiries [17].

The *WirtMed study*, funded by the Innovation Fund of the Federal Joint Committee (*Gemeinsamer Bundesausschuss*, grant number: 01VSF17016), deals with the (further) development of different systems to direct and control cost-efficient drug prescriptions in the light of the legal requirements SGB V §12 [18]. This study, as a sub-project of WirtMed, explores how ASHIP-accredited physicians evaluate the WSV [19]. Our research questions were: 1) How do ASHIP-accredited physicians feel about the need to reduce costs? 2) How do they evaluate the WSV? 3) How do physicians experience the transition from the old system (RGP) to the new system (WSV)? 4) What suggestions for improvement of the WSV do the physicians have?

## Methods

### Study design

We conducted semi-structured in-depth interviews and seven focus groups with physicians as part of a formative evaluation. The focus groups aimed to identify new aspects through joint discussion, while the interviews were designed to capture more detail.

The ethics committee of the Friedrich-Alexander University Erlangen-Nürnberg approved the study (file number: 65_19 B). We followed the guideline “Good Research Practice “ by the Deutsche Forschungsgemeinschaft (DFG, *English:* German Research Foundation).

### Recruitment

Physicians were approached by letter by the Bavarian ASHIP in 2019. Inclusion criteria were specialties with high prescription volume, e.g. internal medicine, and overall target achievement of ≥ 90% according to their trend reports in the quarters of 2017. Physicians with continuously lower overall target achievement were interviewed separately in another sub-project. Interested physicians then contacted the Institute of General Practice of the Universitätstklinikum Erlangen (IGP UK-ER). They received an information letter and the declaration of consent. In a personal conversation with NZ, they could clarify possible questions. After they gave written informed consent, participants were invited by the IGP UK-ER for the interviews/focus groups. All physicians that contacted the IGP UK-ER could take part in the study. One physician did not participate because of the online format of the focus group discussions. Since mostly GPs responded after the first letter, in a second and third letter in February and August 2020 only specialists were contacted. The procedure remained the same.

### Setting of the study

Bavaria is the largest of the 16 states of the Federal Republic of Germany and is located in its southeast. With around 13 million inhabitants, it is the second most populous German state. In Bavaria, there are 9,377 general practitioners and 2,030 practicing specialist internists (including, for example, nearly 30% cardiologists) [20, 21]. The average age of physicians in the outpatient sector is 53.97 years.

### Data collection

Researchers from the IGP UK-ER and the Institute of General Practice at Marburg University (pedagogue/psychologist, and pharmacist) developed the guide for the interviews, and focus group discussions and pre-tested them with four physicians. Minor adjustments were made based on the pretest. The interview/focus group discussion guide comprised four topic sections (Additional file 1):

1. General information on the person: Initially, we familiarized ourselves with the physician and their practice by discussing typical patient cases. We asked open-ended questions about the criteria they consider important when selecting medications, and then presented cards to cover any additional information they wanted to share.
2. General information on prescribing behavior: We inquired about their methods of acquiring drug information and their approach to new medications, as well as those initiated by other specialists.
3. Overall situation regarding WSV: We presented the physicians with a brief article on the introduction of the WSV and proceeded to inquire about their personal experiences. This segment of the survey delved into various areas, such as the advantages and disadvantages of the new system compared to the previous one, the perceived impact on treatment freedom, changes in prescribing behavior, and how physicians manage to meet their targets, among others. Additionally, we asked about their level of satisfaction, their expectations from ASHIP (abbreviated form of an organization), and what would facilitate the prescribing process for them.
4. Conclusion: In the end, participants were given the opportunity to address any other aspects they wished to discuss.

The focus groups followed the same procedure, with the exception of omitting some sub-questions. Additionally, at the beginning of the session, a round of introductions was conducted to ensure that each participant could be associated with a voice recording (detailed instructions can be found in the appendix of the guide).

From 10/2019 to 10/2020 data collection took place. We first conducted all in-depth interviews (average 60 min), followed by focus group discussions (about 120 min). The in-depth interviews were conducted by two female researchers in each physician’s practice. NZ was the main interviewer at every interview. The interviews were digitally audio-recorded. The focus group discussions took place at the IGP UK-ER (focus group 1), in a rented room (focus group 2) or online via WebEx by Cisco or Zoom with the start of the COVID-19 pandemic (focus groups 3–7). NZ (moderator) and JG or MS (co-moderators) led the focus group discussions. The focus group discussions were digitally audio recorded, in case of a video conference only the audio track was saved. In addition, protocols of the sessions were written during and after the in-depth interviews/ focus group discussions. In the protocols, we noted additional information about the interview situation (e.g. disturbances). Interviewers and physicians did not know each other beforehand.

### Data analysis

The audio files were completely transcribed, and subsequently anonymized following a pre-defined protocol [22]. The transcription was done by an external provider. Afterwards, NZ checked all transcripts twice and corrected any errors. Transcripts were not provided to participants. Analysis started when the first data were collected. The methodology followed Qualitative Content Analysis to structure the structure the extensive amount of data. We followed a deductive-inductive procedure, using the software program MAXQDA Plus 2020 [23]. To familiarize with the data, NZ and MS read independently through the transcripts and wrote memos and brief summaries. A system of categories was formed based on the main criteria of the in-depth interview/focus group discussion guide which was deductively applied to the data (e.g. timely feedback from the ASHIP). In the course of the analysis, the category system was inductively expanded by new categories if necessary (e.g. Indifference towards the WSV due to older age of physician). With each new category, we went back into previously coded material to see if any information had been overlooked. For the focus group discussions, new categories have been added inductively after the WSV was revised (version 2.0, and 3.0). All data material was finally coded using the final category system (Additional file 2). The reporting of results follows these categories.

### Quality assurance

NBD, TK and MS have extensive knowledge in qualitative research. Before data collection, NZ and JG have been trained in qualitative data collection.

NZ and MS coded ten percent of the transcripts individually and resolved differences through discussions. All categories were continuously discussed by the project team, meeting the standard of consensual validation. After coding half of the interviews, no new categories were added, indicating saturation of information. During the study, a research diary was kept in which NZ noted her own role and possible influence during data collection, but also initial ideas for the analysis. The content of the research diary was regularly discussed with MS].

A communicative validation of the preliminary results took place with teaching and further training physicians affiliated with the IGP UK-ER within the framework of the "Day of General Practice/Further Training/Teaching" on 10.10.2020. There, the initial analysis results were visualized and validated with Bavarian General Practitioners (GPs). They were not former participants. The reporting of the study is following the "Consolidated criteria for reporting qualitative research" (COREQ) [24]. Quotations from throughout the data material will be presented. The quotations are named according to the type of data collection (IDI: in-depth interview, FGD: focus group discussion), occupation (GP, specialist), ID and paragraph.

## Results

### Study population

In total, fifty-six physicians (40 men, 16 women) in age groups ranging from 41 to over 60 years participated in the study (Table 1). Of these, twenty participated in an in-depth interview (GPs: *n* = 18; specialists *n* = 2) and 36 physicians participated in one of seven focus group discussions. In the first two focus group discussions (n_1_ = 8, n_2_ = 2) only GPs participated. In the following 5 focus group discussion, mostly specialists participated (n_3_ = 3; n_4_ = 5; n_5_ = 4; n_6_ = 7; n_7_ = 7). There were different specialties represented from internal medicine (pulmonology, cardiology, (hemato)oncology, rheumatology, gastroenterology, nephrology), GPs, neurologists/psychiatrists, and a surgeon. Different medical practice types, such as single, or group medical practices, as well as various locations (rural, small/medium town, large city) were represented.

**Table 1:** Sample characteristics

	Total (*N* = 56)
	*n*
Gender	
Male	40
Female	16
Specialist field	
General Practitioners	30
Pulmonologist	5
Cardiologist	5
Neurologist/Psychiatrist	4
(Haemato-)Oncologist	4
Rheumatologist	3
Gastroenterologist	2
Nephrologist	1
General internist	1
Surgeon	1
Age group	
30 – 40 years	2
41 – 50 years	17
51 – 60 years	20
over 60 years	16
Practice type	
Single practice	28
Group practice	20
Other^a^	8
Practice location	
Rural area^b^	8
Small/medium town^c^	29
City^d^	19
Employment type	
Full-time	25
Part-time	3

^a^Other = Group practice, Individual practice with employed physicians

^b^Rural area = < 5.000

^c^Small tow*n* = 5.000 to approx. 20.000, Medium tow*n* = 20.000 to approx. 100.000

^d^City = 100.000 or more inhabitants

### The need to reduce costs

Physicians saw the necessity and duty of acting economically; however, they did not think that everyone shared this mindset:

"*Costs are important […]. As an individual, you also have responsibility for the […] solidarity system.*" (IDI-GP-03:283).

For some, economic considerations even took precedence over medical considerations:

"*In the end, everything is subordinated to economic efficiency. Both medical expediency and innovation ultimately, because you can't prescribe anything new without exposing yourself to the risk of sanctions*” (IDI-GP-07:79).

The requirement of economic efficiency could lead to tensions.

"*We […] are the buffer between high demands, demanding patients, demanding guidelines on the one hand. And on the other hand, there are economic constraints. I find myself in a dilemma every day*." (FGD6-specialist-44:179).

However, this priority of cost efficiency was not shared by all physicians:

“*I also think that it is not our job to examine economic efficiency […] And I don't feel responsible for the fact that the costs of drugs are generally too high […].*" (FGD3- specialist-32:149).

### Evaluation of WSV

The physicians mentioned both, positive and negative points. Positive and negative statements cannot necessarily be attributed to one individual but rather overlap (Table 2).

**Table 2:** Main, Subcategories, and derivations of physicians’ WSV evaluation

Main categories	Subcategories	Derivations
Positive aspects of WSV^a^	Timely feedback	The introduction of WSV offers only advantages compared to the prescribing target scheme
	Easy to understand	
	Time-efficient	
	Stimulating for self-reflection	
	More control possibilities	
	More justice	
	Confidence/Trust in agreement	
Negative aspects WSV	Drug discount contracts	Negative evaluation increased when active substance agreement 3.0 was introduced
	Less therapy sovereignty	
	Lack of transparency	
	Fear of sanction	
	Inhibition of innovative drugs	
	No guideline orientation	
	The basic structure of trend reports	
	Concern about successor	

^a^WSV (Wirkstoffvereinbarung, English: Active Substance Agreement)

### Positive aspects: Physicians attribute many advantages to the WSV

The physicians were often positive in their overall assessment of the WSV 2.0 and could imagine this model for the future.

*“[The WSV 2.0] would be a model that I could also imagine for the future. [...] There is a target. If it is not set too high and is realistic and at the same time protects us from unnecessary sanctions, I think it is a good way for all parties involved.”* (FGD5-specialist-42:130)

The positive aspects were weighted significantly more heavily, while criticism was primarily broadly based.

Physicians perceived more control options due to early feedback in form of the trend reports:

"*We get the data promptly. […] . In the end, we have quarterly data, so that we can also take countermeasures very quickly - which we didn't have before.*" (FGD4-GP/specialist-35:228).

The visual representation in the form of traffic lights served as an impulse for the physicians; also, to seek advice from the ASHIP’s pharmacotherapy consulting (IDI-GP-04:85). The trend reports inspired physicians to self-reflection:

"*We take it as a rationale for our actions, but we can't implement it one hundred percent.*" (specialist-16:59).

The physicians did not address the trend reports that existed during the RGP, indicating that they did not pay attention to them.

Some stated that generic drugs made prescribing even easier (IDI-GP-17:81). In addition, the comparison with their specialist group helped them to consider whether prescribing differently would "*also [be] more economical*" (IDI-specialist-11:109).

The WSV also ensured more justice according to some physicians and was described as a "*great liberation*" (IDI-GP-06:65). Physicians have a feeling of security and safety: "*There are quite a few safety nets, where you are rescued if you have a red light [in another drug indication group]*.” (FGD1-GP-24:257). One of these safety nets could be ASHIP counseling (IDI-GP-13:69). Many physicians also reported perceiving more therapeutic autonomy after the targets were introduced (IDI-GP-02:258).

It is noteworthy that in particular, GPs expressed great appreciation. Among specialists, opinions varied by specialty; for example, gastroenterologists seemed more satisfied with the WSV than interviewed nephrologists (FGD5-specialist-40:51, specialist-16:61).

### Negative aspects: Physicians still experience issues with the system of the WSV

In the negative evaluation category, the data showed a wide variety of problems with the health care system in general and with WSV in particular. The reported issues were very individual and depended on the specialty of the reporting physician. The criticisms did not differ between WSV versions but intensified during the transition from version 2.0 to 3.0.

As general criticism, some physicians stated that the bureaucratic effort took up more time than the actual patient care itself (FGD6-specialist:181, IDI-GP-07:111). Some physicians felt generally free in their therapeutic autonomy, but e.g. wished for more individual treatment possibilities:

"*What I think is missing is a certain freedom for trying out an individual therapy [such as] [...] Entresto in the field of cardiology. Some patients with mixed diastolic-systolic heart failure [...] might benefit from it. And that is not something that I can prescribe to this group of patients*." (FGD7-specialist-55:203).

This was especially, but not exclusively, reported by older physicians. They reported a lack of certainty concerning the WSV, because of potential changes in the future (FGD3-specialist-32:201). Some reported a lack of transparency and own understanding.

Physicians feared that restrictive prescribing behavior by colleagues could ultimately lead to stricter targets with the next WSV revision, as the impression could be created that the targets are easy to meet (IDI-GP-13:150). Few specialists noted that the system is not fully capable of addressing the complexity of daily practice (e.g. no specifications for every kind of kidney disease). At the same time, however, the physicians pointed out that the system is nevertheless good and should be retained; not least because it is suitable for most specialties (IDI-specialist-16:61). When handling drug prescriptions initiated by specialists, patients sometimes are referred elsewhere in fear of potential sanctions; despite raising costs for the system overall due to repeated examinations.

Some of the targets were perceived as outdated, and physicians wished for more flexibility such as in the handling of direct oral anticoagulants (IDI-GP-20:89). Others, in contrast, preferred stability to continuous regulatory changes (IDI-GP-03:153, FGD5-specialist-42:199). Few physicians perceived a mismatch between WSV and medical guidelines, which would hinder the prescribing of drugs that the physicians considered innovative (FGD4-specialist-38:128, FGD4-GP/specialist-35:123–129). This refers to certain medications such as oral anticoagulants as there are different recommendations between the German and European guidelines and the WSV refers to the German guidelines for GPs (IDI-GP-13:45).

Criticism that was mentioned often by physicians regardless of specialty was the existence of drug discount contracts (IDI-GP-01:141, FGD3-specialist-32:221, FGD5- specialist-40:60). The physicians noted that the pharmaceutical industry still exerts great influence through drug discount contracts. Physicians mentioned the topic on their own and reported various difficulties concerning such contracts which are listed in Table 3.

**Table 3:** Physicians’ difficulties with drug discount contracts

Drugs from discount contracts are…
… difficult for patients, who consequently often receive drugs from other pharmaceutical companies. Consequences might be mix-ups of medications, double intake, or no intake at all
… difficult for physicians due to additional work (search in the system for discounted drugs; additionally, dependent on the health insurance company)
… non-transparent and as the physicians perceive it as not reasonable (cost–benefit ratio)

### Transition from the old system (RGP) to the new system (WSV)

The physicians consistently perceived the WSV as an improvement compared to the previous RGP.

“I find it [the WSV] much clearer compared to the previous system [RGP].” (FDG1-specialist-26:452)

Especially the introduction of the trend reports was seen as innovative, the feedback was easy to understand, and thus time-efficient (IDI-GP-08:390, IDI-GP-14:43).

The WSV was intended to create a control instrument that would take effect at an early stage so that sanctions would not become necessary. This goal was achieved for some physicians and fear of sanctions had decreased (IDI-GP-15:16; FGD7-specialist-56:101). Others were still fearful of sanctions (IDI-GP-04:89, IDI-GP-10:75).

Problems in the transition were primarily in the communication with ASHIP and the transparency of the regulations. Some statements show a lack of information about the WSV. For example, some physicians thought that prescribing new drugs in principle results in missing their targets.

It is striking that for some physicians the transition to a new system did not elicit any reaction. They were somewhat indifferent to the current system (IDI-specialist-11:31). When their prescriptions were rated as economically efficient anyway, they perceived the changes in the system as not relevant to them (IDI-GP-02:162). Some older physicians reported that they developed a relaxed attitude due to their experience with regulation changes in the system and welcomed the trend reports (GP-19:36). At the same time, younger GPs also claimed there could be differences between generations; older colleagues would criticize the system more than younger colleagues, as the latter would already be used to adhering to strict economic guidelines. The work experience could therefore lead to a de-stressed or stressed attitude.

Satisfaction with WSV 2.0 was high and decreased during the transition to the adjusted system of WSV 3.0. The physicians reported significantly harder targets that are difficult to reach:

*“We used to be in the 100 percent range and now we're slipping to 80. All of a sudden you're in the orange range without us changing anything in our prescribing behavior.”* (FGD5-specialist-41:54)

The transition to WSV 3.0 was therefore much more difficult.

### Suggestions for future regulations

Despite the overall positive evaluation, all physicians contributed suggestions for improvement. The suggestions can be divided into three sections: 1. improvements in communication on the part of the ASHIP (content, and format). 2. improvements in the WSV drug regulation scheme, and 3. development of the trend reports.

1. Improvements in communication on the part of the ASHIP: Physicians would like to receive more explanations on changes in trend reporting, information on which guidelines are WSV-compliant, and a pharma-independent drug assessment. Physicians often reported that pharma representatives play little or no role in their practice (e.g. FGD1-GP-28:156, FGD6-specialist-43:154). Regarding the format, many found the counseling with the ASHIP pharmacologists helpful, as well as the introductory training for new physicians. Some specialists, on the other hand, also reported that the ASHIP counseling was not very helpful concerning specific medications. They wished for a different information transfer instead of e-mails. According to the interviewed physicians, the official website should be more user-friendly so that important information on medications could be accessed quickly.
2. Improvements in the drug regulation scheme as a whole: Physicians would tendentially rather have loose guidelines instead of strict instructions. According to a few, physicians’ comparison groups should be fitted more appropriately by the ASHIP (IDI-GP-13:101). Physicians asked for more transparency on criteria development and would like to be involved in the development of criteria. Some physicians demanded more flexibility and exceptions (e.g., severity staging). Some would like a more dynamic adaptation to new circumstances, others preferred more stability. Suggestions concerning drug discount contracts ranged from abolishing vs. standardizing them (across health insurers), and across packaging sizes. If drug discount contracts are unavoidable, then they should at least not apply to generics and be transparent in terms of pricing.
3. Improvements in trend reporting: The traffic light in the trend reports should be retained, with the possibility of error reporting to the ASHIP. Further breakdowns were wished for, such as a comparison of similar practices (e.g. in terms of location) or the percentage impact of a target value on the overall result.

## Discussion

### Study population

The sample size of 56 physicians is not representative, although this is not the goal of qualitative studies. The size is considerable for a qualitative study and aligns with many aspects of the medical profession in Bavaria. In Bavaria, there are 9,377 general practitioners and 2,030 practicing specialist internists (including, for example, nearly 30% cardiologists) [20, 21]. In our study, there are proportionally more general practitioners who were interviewed (54% GPs vs. 46% specialists). The physicians in Bavaria have an average age of over 50 years [25], which is also true for the participants in our study. Furthermore, the medical profession is predominantly male, and we also interviewed more male physicians.

### The need to reduce costs

The physicians see the need to reduce costs unlike previous studies reported [26]. Compared to the previous system, timely feedback helps physicians to have more control over their prescribing situation. Perceptions of the participants ranged from very positive evaluations, such as fewer sanction threats, to more negative perceptions, such as less therapeutic autonomy. However, the positive aspects far outweighed the negative ones. A variety of suggestions for improvement could be elicited, e.g. participatory development of targets for generics or lead substances, (even) more transparency regarding criteria development, and changing the system of drug discount contracts.

### Evaluation of WSV

The WSV seems to be the reason for the largely reduced sanction concerns of physicians. The old cost-based system led physicians—especially GPs—into sanctions [27]. Drug discount contracts, however, were strongly criticized by physicians but played a big role in reducing drug costs in Germany. Other studies also confirm that the frequent substitution of different generic drugs according to the latest discount contract extends the need for discussion and explanations with patients to avoid possible mistakes in taking medications [28]. When drugs are handed out in the pharmacy, physicians do not know which specific tablets the patients receive. This means that patients receive the prescribed active ingredient, but from different pharmaceutical companies/manufacturers, depending on the drug discount contracts currently in place [28, 29]. Some physicians felt that the potential cost savings may not be significant. This is contradicted by the fact that some drug discount contracts have been published, which showed that health insurance companies received discounts of up to 99% in some cases [30]. However, follow-up costs of medication mix-ups or intake errors might be considered as an older study suggests [28]. Physicians demanded more information concerning the WSV and drug discount contracts. The influence of the pharmaceutical industry on prescribing behavior has been investigated in numerous studies [31]. The physicians were critical of the influence of the pharmaceutical industry. Even the influence over the negotiation of the drug discount contracts was still too great for them.

### The transition from the old system (RGP) to the new system (WSV)

In the past, physicians in Germany were able to prescribe with significantly less regulation but with more severe consequences in case of sanctions. Nearly all physicians noted early feedback from the trend reports, although feedback already existed under the RGP [32]. It seems that physicians are more aware of them under the WSV; maybe due to the traffic light system. The physicians described the trend reports as a safety net that allows them to react early. In other studies, regular feedback on prescription/costs lead to a reduction in expenditure [33]. The extent to which the WSV will reduce costs will be part of further analysis.

Some physicians felt indifferent and showed no interest in the WSV. However, this does not necessarily mean that they prescribe uneconomically. They were either focusing on the economic efficiency principle regardless or reportedly said it is due to their age and/or years of experience with former cost regulation system changes. This is also reported in other international studies, e.g. general practitioners in Iran and Denmark [34, 35]. In the former, older physicians, as well as very young ones with less experience and females, are less economically efficient. In our interviews, there were no suggestions that female physicians could be less cost-efficient. In terms of age, the latter study suggests that older physicians may have achieved their own desired financial income goals [35]. Aging might lead to a reduced willingness and ability to perform strenuous work like dealing with drug regulation systems in some cases [36, 37].

### Suggestions for future regulations

Concerning the suggestions for improvement, some physicians asked for more transparency regarding the development of drug targets, and/or even co-development with themselves. However, this transparency is already in place but is not realized by physicians as such. Misinformation among the physicians was also evident. Some physicians did not prescribe new drugs in principle, thinking it would result in not meeting their targets in certain indication groups. In reality, newer drugs that show relevant advantages compared to commonly used medications can potentially be prescribed. That means in turn, other information channels might be considered in the future to avoid such misinformation some physicians have. The WSV aims to allow physicians to focus more on medically correct prescribing than on prescribing economics [6]. Nevertheless, the medical practices of physicians today are also a business enterprise that, if regulated by legal authorities and laws such as §12 SGB V, must be supplied with information. The Bavarian ASHIP and sick funds worked on the WSV to comply with their legal obligations and prevent an audit.

Misaligned incentives in the system should be minimized as far as possible. On the one hand, there are financial incentives for an excess of medical services and, on the other hand, there are budgeting measures that prevent all patients from receiving sensible and necessary treatment [38, 39]. In the future, a comparison of European regulatory systems could be of great interest to identify further potential for improvement.

### Limitations and future research

This study subproject of the WirtMed study aimed to analyse the perception of physicians that have a total target achievement of ≥ 90%. Physicians who prescribed uneconomically over several quarters were not interviewed here and are part of another subproject of the WirtMed study. It is possible that the doctors who achieve better results also rate the WSV better. However, it is important to note that already a target achievement < 100 can lead to sanctions for physicians and it is thus not the case that we have only included physicians in our study who perform well.

When recruiting specialists, the quarter 04/2019 was inadvertently used instead of the quarters 01/2017 to 04/2017 for specialists with a total target achievement of ≥ 90%. In the interviews, the trend reports were presented or communicated verbally by the physicians themselves. They were not analyzed independently in terms of content. Any qualitative study reflects the subjective perceptions of the interviewed physicians and can elicit a range of opinions. Future research will include a quantitative evaluation of changes in cost expenditures as part of the larger WirtMed study.

## Conclusion

In 2014, the Bavarian sick funds implemented a new system to reduce costs by introducing the WSV in Bavaria. In our study, we explored qualitatively how ASHIP-accredited physicians evaluate the WSV and which suggestions for improvement of the WSV they have [19].

The size of the sample is substantial for a qualitative study and aligns with many aspects of the medical profession in Bavaria such as specialty field, age, and gender. The physicians in the study recognize the need to reduce costs and appreciate the timely feedback provided by the system. They have varying perceptions of the system, with some positive evaluations such as fewer sanctions threats, but also concerns about reduced therapeutic autonomy.

Physicians’ suggestions for improvement include participatory development of targets for generics, increased transparency in criteria development, and changes to the drug discount contract system.

The implementation of the WSV appears to be the primary reason for reduced sanction concerns among physicians. However, drug discount contracts received criticism despite their role in reducing drug costs. The influence of the pharmaceutical industry on prescribing behavior is a concern for physicians, and they believe that the industry still has significant influence over the negotiation of drug discount contracts.

The transition from the previous system to the WSV has increased physicians' awareness of feedback and trend reports, introducing them to an early safety net for reacting to prescribing patterns. Some physicians expressed indifference or lack of interest in the WSV, attributing it to their focus on economic efficiency or their experience with previous cost regulation system changes.

Suggestions for future regulations include increased transparency in the development of drug targets and involving physicians in the co-development process. Misinformation among physicians and the need for alternative information channels were also highlighted. Minimizing misaligned incentives in the system and conducting comparative studies of regulatory systems in Europe are potential areas for improvement. Limitations of the study include the subjective perceptions of the interviewed physicians, the exclusion of physicians with poor target achievements, and the use of a specific time period for recruiting specialists.

### Implications for policy and practice

The study shows that policymakers, healthcare professionals, and stakeholders should work together to optimize cost-reduction strategies, minimize industry influence, improve physician engagement, and ensure a balance between economic efficiency and quality patient care.

### Supplementary Information

**Additional file 1.** Interview and Focus Group Guide. Complete guide that was used during interviews and focus groups.

**Additional file 2.** Excerpt from the coding tree. File shows relevant categories, subcategories and descriptions.

## Data Availability

The data that support the findings of this study are available from the corresponding author upon reasonable request.

## Abbreviations

ASHIP, Bavaria: Association of Statutory Health Insurance Physicians, Bavaria
IGP UK-ER: Institute of General Practice of the Universitätstklinikum Erlangen
RGP: Richtgrößenprüfung (English: Prescribing Target Scheme)
SGB V: Sozialgesetzbuch V (English: German Social Code, Book Five)
WSV: Wirkstoffvereinbarung (English: Active Substance Agreement)

## References

[1] OECD. Pharmaceutical expenditure 2020. OECD Publishing. 2020. https://data.oecd.org/healthres/pharmaceutical-spending.htm. Accessed 10 Oct 2022.

[2] Ess SM, Schneeweiss S, Szucs TD (2003). European healthcare policies for controlling drug expenditure. Pharmacoeconomics.

[3] Niehaus F, Finkenstädt V. Deutschland - ein im internationalen Vergleich teures Gesundheitswesen? [Germany - an expensive health care system in international comparison?] Wissenschaftliches Insitut der PFV (WIP). 2009. http://www.wip-pkv.de/fileadmin/DATEN/Veroeffentlichungen/Deutschland_ein_teures_Gesundheitswesen.pdf Accessed 10 Oct 2022.

[4] Korzilius H (2019). Deutsches Gesundheitssystem: Hohe Kosten, durchschnittliche Ergebnisse [German health system: high costs, average results.]. Dtsch Arztebl Int.

[5] GKV-Spitzenverband. Kennzahlen der gesetzlichen Krankenversicherung [Key figures of the statutory health insurance] 2021. https://gkv-spitzenverband.de/media/grafiken/gkv_kennzahlen/kennzahlen_gkv_2021_q2/20210901_GKV_Kennzahlen_Booklet_Q2-2021_300dpi_barrierefrei.pdf. Accessed 10 Oct 2022.

[6] KVB. Steuern statt prüfen - zwei Jahre Bayerische Wirkstoffvereinbarung [Control instead of audit - two years of Bavarian active substance agreement]. 2016. https://www.kvb.de/fileadmin/kvb/dokumente/Presse/Publikation/KVB-FORUM/Einzeldateien-FORUM/2016/KVB-FORUM-11-2016-Titelthema-Wirkstoffvereinbarung.pdf. Accessed 10 Oct 2022

[7] Bundesministerium für Gesundheit. Richtgrößen und Wirtschaftlichkeitsprüfung [Benchmarks and performance audit]. 2016. https://www.bundesgesundheitsministerium.de/service/begriffe-von-a-z/r/richtgroessen-und-wirtschaftlichkeitspruefung.html. Accessed 10 Oct 2022

[8] Osterloh F (2012). Praxisbesonderheiten: Das GKV-Versorgungsstrukturgesetz will die Regelungen vereinheitlichen [The Statutory Health Insurance Structure Act aims to unify the regulations]. Dtsch Arztebl Int.

[9] Weinrich C (2016). Überblick über die künftigen Wirtschaftlichkeitsprüfungen [Overview of future performance audits]. Diabetes aktuell.

[10] Stadhouders N, Kruse F, Tanke M, Koolman X, Jeurissen P (2019). Effective healthcare cost-containment policies: A systematic review. Health Policy.

[11] Kassenärztliche Bundesvereinigung (KBV). Arzneisteuerung und Wirkstoffvereinbarung: Verordnungsempfehlungen zur Zielerreichung [Drug control and active substance agreement: Prescribing recommendations to achieve the 2020 target]. 2020. https://www.kvb.de/verordnungen/arzneimittel/wirkstoffvereinbarung/. Accessed 10 Oct 2022.

[12] Hofmann H, Schöffski O (2008). Generika und Biosimilars [Generics and biosimilars].

[13] Stich V (2007). Verordnungen: Die richtige Dosis finden [Prescriptions: Finding the right dose]. Bayer Aztebl.

[14] Dietz U (2008). Kurze Geschichte der Arzneimittel Rabattverträge und Mutmaßungen über die weitere Entwicklung [Brief history of the pharmaceutical drug rebate contracts and speculations about the further development]. Gesundheits-und Sozialpolitik.

[15] Gröber-Grätz D, Gulich M (2010). Impact of drug discount contracts on pharmacies and on patients’ drug supply. J Public Health.

[16] Kassenärztliche Vereinigung Bayerns. Wirkstoffvereinbarung vom 01.10.2017 [Active substance agreement from 10/01/2017]. 2017. https://www.kvb.de/fileadmin/kvb/dokumente/Praxis/Rechtsquellen/S-Z/KVB-RQ-Wirkstoffvereinbarung-2017.pdf. Accessed 10 Oct 2022.

[17] Kassenärztliche Vereinigung Bayerns. Wirkstoffvereinbarung - Wichtige Änderungen der Wirkstoffvereinbarung zum 01.10.2017 [Active substance agreement - Important changes to the active substance agreement as of 10/01/2017]. 2017. https://www.kvb.de/fileadmin/kvb/dokumente/Praxis/Verordnung/Sonstiges/KVB-Merkblatt-WSV-Aenderungen-ab-2017.pdf. Accessed 10 Oct 2022.

[18] Gemeinsamer Bundesausschuss (G-BA). WirtMed - Die Verordnung von Arzneimitteln: Prüfung und Steuerung von Wirtschaftlichkeit und Qualität (WirtMed-Studie) [WirtMed - The prescription of drugs: Examination and Control of Efficiency and Quality (WirtMed Study)]. 2019. https://innovationsfonds.g-ba.de/projekte/versorgungsforschung/wirtmed-die-verordnung-von-arzneimitteln-pruefung-und-steuerung-von-wirtschaftlichkeit-und-qualitaet-wirtmed-studie.133. Accessed 10 Oct 2022.

[19] Deutsches Register Klinischer Studien. Die Verordnung von Arzneimitteln: Prüfung und Steuerung von Wirtschaftlichkeit und Qualität (WirtMed-Studie) Teilprojekt C: Auswirkungen der Bayerischen Wirkstoffvereinbarung auf der Mikroebene. DRKS-ID der Studie: DRKS00019820 [German Clinical Trials Registry. The prescription of drugs: Examination and Control of Efficiency and Quality (WirtMed Study) Subproject C: Effects of the Bavarian Agreement on Active Substances at the Micro Level.]. 2019. https://www.drks.de/drks_web/navigate.do?navigationId=trial.HTML&TRIAL_ID=DRKS00019820.

[20] KVB. Versorgungsatlas Hausärzte [Care Atlas of General Practitioners]. 2023. https://www.kvb.de/fileadmin/kvb/V10/Ueber-uns/Versorgungsforschung/Versorgungsatlas/KVB-Versorgungsatlas-Hausaerzte.pdf. Accessed 15 Jun 2023.

[21] KVB. Versorgungsatlas Fachärztlich tätige Internisten [Care atlas of internists]. 2023. https://www.kvb.de/fileadmin/kvb/V10/Ueber-uns/Versorgungsforschung/Versorgungsatlas/KVB-Versorgungsatlas-fachaerztl-taetige-Internisten.pdf. Accessed 15 Jun 2023.

[22] Dresing T, Pehl T. Praxisbuch Interview, Transkription & Analyse, Audio transcription [Interview Practice Book, Transcription & Analysis, Audio Transcription]. 2018. https://www.audiotranskription.de/wp-content/uploads/2020/11/Praxisbuch_08_01_web.pdf.

[23] Mayring P. Qualitative Inhaltsanalyse [Qualitative content analysis]. Handbuch qualitative Forschung in der Psychologie. Wiesbaden: Springer; 2010. p. 601–13.

[24] Tong A, Sainsbury P, Craig J (2007). Consolidated criteria for reporting qualitative research (COREQ): a 32-item checklist for interviews and focus groups. Int J Qual Health Care.

[25] Baye­ri­sche Landes­ärz­te­kam­mer. Presseinformation. Zahlen, Daten, Fakten - Jahresbilanz der Bayerischen Landesärztekammer (BLÄK) zum 79. Bayerischen Ärztetag in München [Bavarian Medical Association. Figures, Data, Facts - Annual Balance Sheet of the Bavarian Medical Association (BLÄK) on the occasion of the 79th Bavarian Medical Congress in Munich] [press release]. München.

[26] Allan GM, Lexchin J, Wiebe N (2007). Physician awareness of drug cost: a systematic review. PLoS Med.

[27] Kassenärztliche Vereinigung Bayerns. Die aktuelle Wirtschaftlichkeitsprüfung: Willkür bei den Kriterien. Leitfaden und Analysen zur Unterstützung der Hausärzte bei Arzneimittelregressen und Prüfverfahren [The current Performance Audit: Randomness in criteria. Guidance and analyses to support primary care physicians in drug reviews and audit procedures]. 2012. https://www.kvb.de/fileadmin/kvb/dokumente/Praxis/Infomaterial/Verordnung/KVB-Broschuere-Wirtschaftlichkeitspruefung.pdf. Accessed 10 Oct 2022.

[28] Leutgeb R, Mahler C, Laux G, Szecsenyi J (2009). Krankenkassen-Rabattverträge: Probleme und Risiken für den Hausarzt bei der Betreuung chronisch kranker Patienten [Health insurance discount contracts: Problems and risks for primary care physicians in the care of chronically ill patients]. DMW-Deutsche Medizinische Wochenschrift.

[29] Quinzler R, Bertsche T, Szecsenyi J, Haefeli WE (2008). Teilung von Tabletten: Welchen Einfluss haben die Rabattverträge auf die Verordnungsqualität? [Division of tablets: What impact do rebate contracts have on prescription quality?]. Med Klin.

[30] Deutsches Aerzteblatt. Datenpanne: Hersteller gewährt mehr als 99 Prozent Rabatt auf Generika [Data breach: Manufacturer offers more than 99 percent discount on generics] Dt. Aerzteblatt: Dt. Aerzteblatt; 2021. https://www.aerzteblatt.de/nachrichten/127917/Datenpanne-Hersteller-gewaehrt-mehr-als-99-Prozent-Rabatt-auf-Generika. Accessed 10 Oct 2022.

[31] Fickweiler F, Fickweiler W, Urbach E (2017). Interactions between physicians and the pharmaceutical industry generally and sales representatives specifically and their association with physicians' attitudes and prescribing habits: a systematic review. BMJ Open.

[32] Kassenärztliche Vereinigung Bayerns. Prüfungsvereinbarung 2009 - 2015 [Audit Agreement 2009–2015]. 2015. https://www.kvb.de/fileadmin/kvb/dokumente/Praxis/Rechtsquellen/Pruefung/KVB-RQ-Honorarpruefung-Pruefungsvereinbarung-2009.pdf.

[33] Yarbrough PM, Kukhareva PV, Horton D, Edholm K, Kawamoto K (2016). Multifaceted intervention including education, rounding checklist implementation, cost feedback, and financial incentives reduces inpatient laboratory costs. J Hosp Med.

[34] Hajibagheri R, Lotfi F, Bayati M (2020). The Efficiency of self-employed general practitioners and factors affecting it: a study in Iran. BMC Res Notes.

[35] Olsen KR, Gyrd-Hansen D, Sørensen TH, Kristensen T, Vedsted P, Street A (2013). Organisational determinants of production and efficiency in general practice: a population-based study. Eur J Health Econ.

[36] van den Berg T, Elders L, de Zwart B, Burdorf A. The effects of work-related and individual factors on the Work Ability Index: a systematic review. Occup Environ Med. 2009;66:211–20. 10.1136/oem.2008.039883 19017690

[37] Alcântara MA, Sampaio RF, Assuncao AA, Silva FCM (2014). Work Ability: using structural equation modeling to assess the effects of aging, health and work on the population of Brazilian municipal employees. Work.

[38] Blumenthal S, Donner-Banzhoff N, Popert U, Kühlein T. Zwischen Heilkunst und Kommerz: Welche Ökonomie verträgt „gute “Medizin? [Between the art of healing and commerce: What kind of economy can "good" medicine tolerate?]. Zeitschrift für Allgemeinmedizin. 2021;97:66–71.

[39] Ankowitsch E (2013). Arzt-Patient-Beziehung: Vertrauen über Jahrzehnte weggespart [Physician-patient relationship: Trust stripped away over decades]. Dtsch Arztebl Int..

