# Supplementary material for: Physicians’ assessment of the Bavarian drug-expenditure control system: a qualitative study
Source: BMC Health Serv Res. 2023 Sep 7;23:961. doi: 10.1186/s12913-023-09844-3 (PMC10483772; doi:10.1186/s12913-023-09844-3)
Supplement: Supplementary file 2 — Additional file 2. Excerpt from the coding tree. File shows relevant categories, subcategories and descriptions. [file 12913_2023_9844_MOESM2_ESM.docx]

**Additional file 2**

*Excerpt from the coding tree*

| Main categories | Subcategories | Descriptions |
| --- | --- | --- |
| General attitudes towards the healthcare system | Necessity and duty of acting economically  Attitude towards pharma representatives or companies  The requirement for economic efficiency leads to tensions | Includes attitudes of physicians towards responsibilities of the physician as an individual for the whole system  Includes attitudes of physicians towards being consulted by pharma representatives or participating in training offered by pharma companies  Includes statements about the physician’s standing between economic efficiency and medical considerations |
| Positive evaluation of WSV | Timely feedback | Includes statements of physicians about getting feedback from the ASHIP earlier than before the introduction of the WSV |
|  | Easy to understand | Includes statements of physicians in which they emphasize the comprehensibility of the WSV |
|  |  |  |
|  | Time-efficient | Includes statements of physicians in which they mention the time aspect have been improved towards more efficiency |
|  | Stimulating for self-reflection | Includes statements of physicians that the WSV uses a visual representation to give an impulse for self-reflecting. This also makes comparisons with other physicians in their specialist group possible. |
|  | More control possibilities | Includes statements of physicians about having more control options which reduce the pressure and the fear of sanction |
|  | More justice | Includes statements of physicians emphasizing that WSV is a step forward toward greater justice |
|  | Confidence/Trust in agreement | Includes statements of physicians in which they mention to feel more safely with the WSV because of trust in safety nets |
| Negative evaluation WSV | Drug discount contracts | Includes statements of physicians in which they mention difficulties arising because of drug discount contracts |
|  | Less therapy sovereignty | Includes statements of physicians in which they criticize the limitation of their therapeutic autonomy and restrictions regarding their prescribing behavior by the WSV |
|  | Lack of transparency | Includes statements of physicians concerning problems with clarity and comprehensibility of the WSV |
|  | Fear of sanction | Includes statements of physicians concerning the WSV causes fear of sanction, which leads to sending patients elsewhere if the prescription costs are too high |
|  | Inhibition of innovative drugs | Includes statements of physicians that they are hindered from prescribing drugs perceived as innovative |
|  | No guideline orientation | Includes statements of physicians which criticize that there is a discrepancy between the WSV and medical guidelines |
|  | The basic structure of trend reports | Includes statements of physicians who criticize the objectivity of the basic structure concerning the criteria and the creators of the WSV |
|  | Concern about successor | Includes statements of physicians in which they worry about finding a successor for their practice, because of the difficulties the WSV brings to daily work |
| Abstention due to lack of knowledge and interest | Focus on the efficiency principle  Indifference due to age  Indifference due to regular  regulation changes | Includes statements of physicians in which they mention that they have always prescribed considering the efficiency principle  Includes statements of physicians in which they mention that younger and older GPs differ in their attitude towards the WSV  Includes statements of physicians in which they mention that they developed a relaxed attitude towards the WSV because of the regular regulation changes |
| Improvement suggestions | Communication  General drug regulation scheme  Trend reporting | Includes statements of physicians, in which they request for more explanations of innovations, information on which guidelines are WSV-compliant and a pharma-independent drug assessment  Includes statements of physicians, in which they request for loose guidelines instead of strict instructions and wish for more transparency and involvement in the process of making criteria for WSV  Includes statements of physicians, in which they require the maintenance of the traffic lights and the implementation of the opportunity of error reporting to the ASHIP |
